# The nutritional use of millet grain for food and feed: a review

**DOI:** 10.1186/s40066-020-00282-6

**Published:** 2021-03-29

**Authors:** Z. M. Hassan, N. A. Sebola, M. Mabelebele

**Affiliations:** Department of Agriculture and Animal Health, College of Agriculture and Environmental Sciences, University of South Africa, Pretoria, South Africa

**Keywords:** Health, Feeds, Finger millet, Foods, Nutritional composition, Pearl millet, Phenolic profile

## Abstract

Worldwide, millets are regarded as a significant grain, however, they are the least exploited. Millet grain is abundant in nutrients and health-beneficial phenolic compounds, making it suitable as food and feed. The diverse content of nutrients and phenolic compounds present in finger and pearl millet are good indicators that the variety of millet available is important when selecting it for use as food or feed. The phenolic properties found in millets compromise phenolic acids, flavonoids, and tannins, which are beneficial to human health. Moreover, finger millet has an exceptionally unique, more abundant, and diverse phenolic profile compared to pearl millet. Research has shown that millet phenolic properties have high antioxidant activity. The presence of phytochemicals in millet grains has positive effect on human health by lowering the cholesterol and phytates in the body. The frantic demands on maize and its uses in multiple industries have merited the search for alternative grains, to ease the pressure. Substitution of maize with pearl and finger millets in the diets of different animals resulted in positive impact on the performance. Including these grains in the diet may improve health and decrease the risks of diseases. Pearl millet of 50% or more can be used in broiler diets without adversely affecting broiler performance or egg production. Of late, millet grain has been incorporated in other foods and used to make traditional beverages. Thus, the core aim of this review is to provide insight and comprehension about the nutritional and phenolic status of millets and their impact on human and livestock.

## Background

Millets are cereals from the Poaceae grass family and are considered one of the oldest cultivated crops. Generally, pearl millet (*Pennisetum glaucum*) and finger millet (*Eleusine coracana*) are known as the two major millets used for food and feed. Pearl millet is believed to have originated from sub-Saharan Africa, and finger millet from the sub-humid uplands of East Africa [1]. The two account for most of the world’s millet production and trade [2]. The majority of the recent research and agricultural programmes, which are routed towards the development of millets, have been dedicated to pearl and finger millets. Dube et al. [3] believe that the urge to route for millet and sorghum instead of maize and other major crops in recent years is derived from the fact that these grains are ecologically well-matched with semi-arid areas because of their ability to tolerate drought. They are considered tough crops in terms of growth requirements as they withstand harsh climatic factors such as unpredictable climate and nutrient-depleted soils [4].

Globally, pearl millet is an important grain and is considered the sixth highest producing crop, after maize, wheat, rice, barley, and sorghum [5]. It is also considered one of the crops that can provide good nutrition and income to small-scale farmers [6] and thus, contributes to livelihoods and the availability of food. Despite its value and contribution, pearl millet does not receive the attention it deserves as a crop that has an important role to play in food security. Perhaps the disregard can be attributed to it being termed a crop for poor farmers in marginal agricultural areas affected by socio-ecological conditions [1]. According to several researchers, millets can be an important source of essential nutrients such as amino acids, and mineral and trace elements [7]. Obviously, wide variations should be evident in the nutritional composition of pearl and finger millets [2]. Shweta, [8] reported that pearl millet contains higher energy compared to cereal grains such as rice and wheat, and is considered a significant source of thiamine, niacin, and riboflavin as stated by [9]. Moreover, the content of minerals such as calcium, iron and phosphorus in pearl millet is like those found in other cereals [10].

In addition, finger millet grows better in colder areas that have slightly more rain [11]. In many rural communities in East and Central Africa, finger millet is known as an important cereal as it contributes significantly to nutritional well-being. In spite of its valued role and its vital contribution to food availability of many underprivileged farmers and families in Africa, it is also a crop that is overlooked [12]. The protein found in finger millet is considered as superior as it encompasses vital amino acids such as lysine, threonine, and valine [13].

Millets are also believed to have nutraceutical health benefits. These include but are not limited to, an increase in digestive system well-being, a reduction in cholesterol, the prevention of heart disease, protection against diabetes, the lowering of cancer risks, and an increase in energy levels and improvement of the muscular system [14, 15].

These characteristics ought to place such grains in the right position in terms of alternative crops; however, due to a lack of attention, millet was termed the ‘lost crop’ [2]. Given the current challenges regarding sustainable food production, climatic changes, and water scarcity, coupled with overpopulation, an interest has been developed regarding millet. This has provided an opportunity for farmers, nutritionists, and food and feed manufacturers to engage in research in order to understand the nutritional and functional characterisation of millet grains. Although reviews in this area have been published recently [16], this review is comprehensive and provides important updates on the utilisation of pearl and finger millets in diets for humans and animals. Specifically, this review (1) provides detailed nutritional composition and its benefits to humans and livestock; (2) it summarises the phenolic properties found in pearl and finger millet grains, as well as their contributions to health or as anti-nutritive factors in animal feeding, and (3) it discusses millets in feed and food applications. Thus, the overview and core objective of this review is to provide insights into the selection of millets for different purpose to maximise their potential as food and feed.

### Physical characteristics

**Fig. 1:**
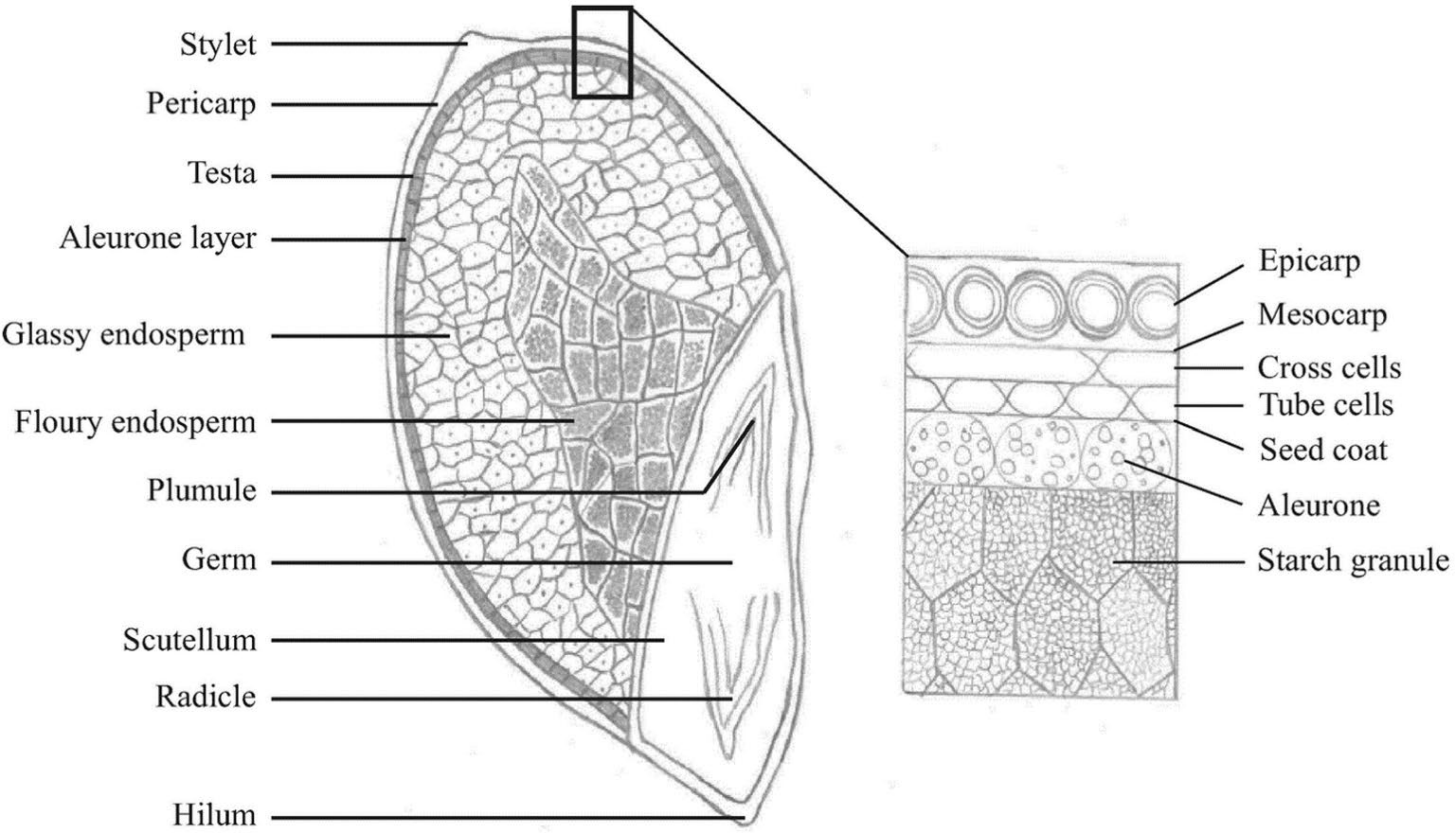
Grain structure of pearl millet

Generally, the kernel structure of different millets is like that of sorghum. It consists of the pericarp, germ, and endosperm (Table 1; Fig. 1). Like with sorghum, the kernel of pearl millet is caryopsis, where the pericarp is entirely attached to the endosperm. However, in finger millets, sack like pericarps are loosely connected to the endosperm at one point. These types of kernels in finger millet are known to be utricle, whereby their pericarp can easily detach for the testa to shield the endosperm [17].

**Table 1:** Anatomic characteristics of millet grains and sorghum

Grain	Type	Shape	Colour	1 000-kernel weight (g)
Sorghum	Caryopsis	Spherical	White, yellow, red, brown	25–30
Pearl millet	Caryopsis	Ovoid, hexagonal, globose	Grey, white, yellow, brown, purple	2.5–14
Finger millet	Utricle	Globose	Yellow, white, red, brown, violet	2.6

Source: 19

As indicated by Abdelrahman et al. [18], the relative distribution of the pearl millet is 8.4% of the pericarp, 75% of the endosperm and 16.5% of the germ [18]. Thus, the proportion of the pearl millet’s endosperm to germ is about 4.5:1, and 8.4:1 in sorghum. The ratio is smaller in finger millet because of its small germ, namely, the endosperm to germ ratio is 11:1 to 12:1, which is more than sorghum and pearl millet [18]. As indicated in Table 1, variations exist between the visual colour of pearl and finger millets, with the 1000 kernel weight being very small for the finger millet [19]. An endosperm is regarded as the largest part of the cereal grain and it acts as a storage tissue [20]. In millets, “the aleurone layer is a single layer of cells which lies just below the testa” [18]. The texture of the millet kernel is controlled by the size of floury and corneous endosperm. More floury than corneous endosperm is found in soft-textured kernels however, solid kernels have more thickly filled corneous endosperm. Moreover, the endosperm of the finger millet is equally divided between the corneous and floury areas. These types of endosperm are known to have an in-between texture. In pearl millet, wide variations exist among the kernel textures as follows: floury, very soft, corneous, and very hard endosperm**.**

**Fig. 2:**
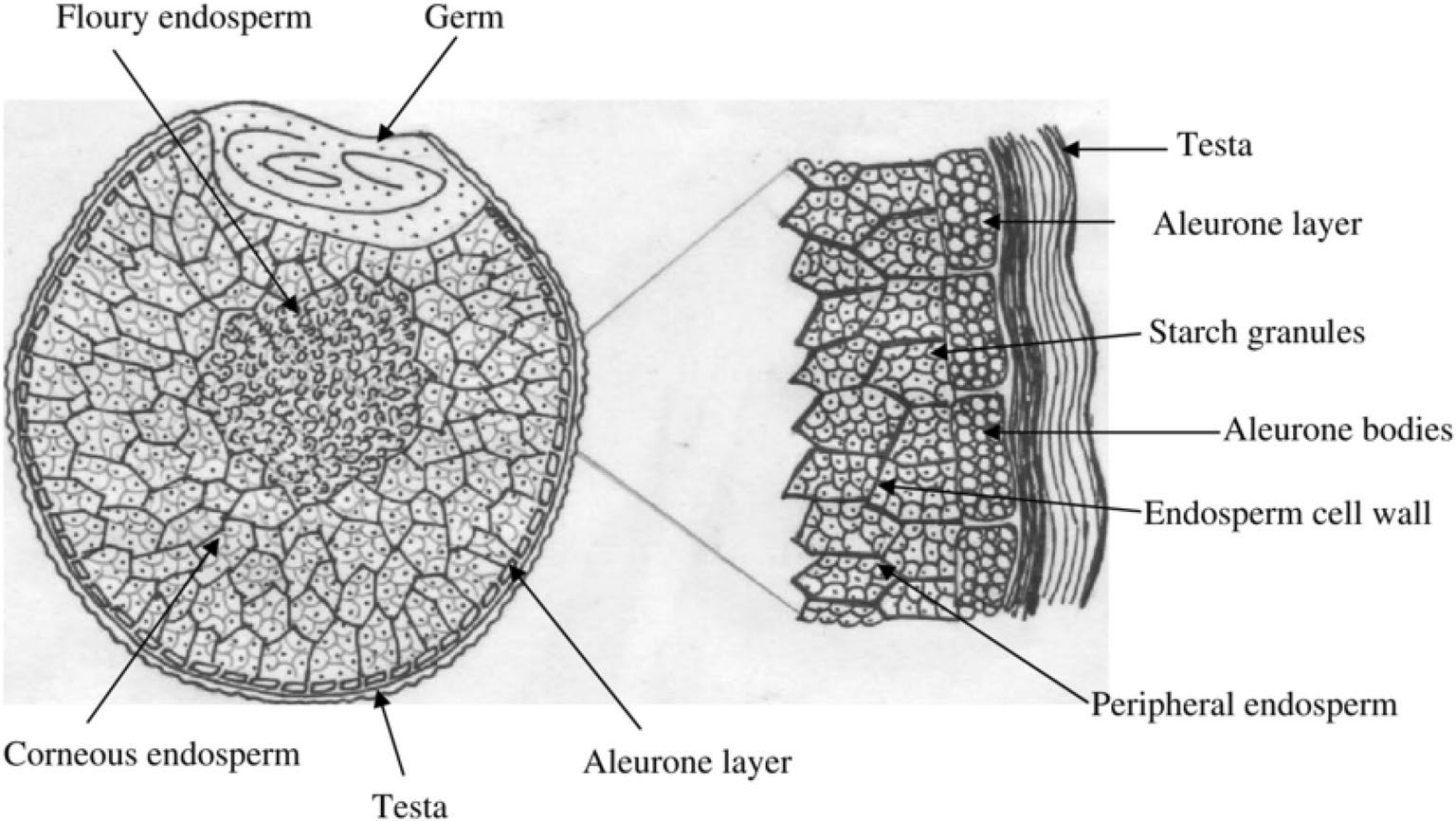
Schematic diagram of a finger millet section. Source: 21

The embryo of finger millet is in a depression enclosed by a distinctive crest and the hilum is located adjacent to the germ [21]. Whilst the protein bodies are small spheres below the cell walls [21] (Fig. 2).

When it comes to the processing and food quality of millets, the texture of the grains is an important contributing factor to consider [22]. There are higher flour yields when dry milling corneous as opposed to soft floury kernel types. Cultivars with higher amounts of corneous endosperm are preferred when making thick porridge. In contrast, the flour produced from soft endosperm is greatly desired [22] when making either fermented or unfermented bread. With animal feeding, an intermediate texture is preferred over the corneous and floury texture as it increase the starch digestion.

### Nutritional profile of millets

Nutritionally, the energy value, protein and macro nutrient contents of millets is comparable and sometimes higher than conventional cereals. They significantly contribute to human and animal diets owing to their high levels of energy, calcium, iron, zinc, lipids, and high-quality proteins. In addition, they are also rich sources of dietary fibre and micronutrients.

### Carbohydrates

The carbohydrates in pearl millet grains incorporate starch, dietary fibre, and soluble sugars. Starch is considered a predominant component of pearl millet endosperm, which comprises glucose in the form of amylase and amylopectin. Various pearl millet grain genotypes vary in starch composition from 62.8 to 70.5% and around 71.82 to 81.02% as reported by [23], soluble sugars range from 1.2 to 2.6% and amylose from 21.9 to 28.8%. However, [24] recorded a low figure of 34.5 and 39.4 g/100 g of starch for pearl millet. The starch in pearl millet can be used as thickening, gelling and as bulking agents for the textural properties of foods [25]. Finger millet on the other hand has a total carbohydrate content that ranges between 72 and 79.5%, as recorded by [26]. Moreover, the detailed profile of the carbohydrates was recorded by [27] to be at a range of 59.5 and 61.2% for starch, 6.2–7.2% for pentosans, 1.4–1.8% for cellulose, and 0.04–0.6% for lignin.

### Proteins

The second major component of millet is protein. Pear millet is believed to contain about 11.6% protein, which is higher than the 7.2% protein found in rice, 11.5% found in barley, 11.1% found in maize and 10.4% found in sorghum which is [28]. In addition, [7] recorded protein content of 9.79% found in pearl millet. In comparison to maize, by weight, pearl millet grain is believed to be about 8–60% higher in crude protein [29]. In contrast, finger millet contains about 5–8% protein [30], 31 recorded the highest protein content for finger millet of about 11%, while [7] recorded a percentage of 6.32% in finger millet. The quality of protein is believed to be a function of the essential amino acids available in protein (Tables 2, 3).

**Table 2:** Proximate analysis of pearl and finger millets (g/100 g)

Nutrients	Pearl millet	Finger millet
Moisture	12.4	7.15–13.1
Protein	11.6–11.8	7.7
Fat/lipids	4.8–5.0	1.8
Minerals	2.2–2.3	2.7
Dietary fibre	11.3	15–22.0
Neutral detergent fibre	9.0	12.7
Acid detergent fibre	3.3	8.7
Carbohydrates	67–67.5	75.0–83.3
Gross energy (MJ/kg)	17.0	15.8
Minerals (mg/ 100 g)		
Phosphorus	296	130–250.0
Potassium	307	430–490
Magnesium	137	78–201
Calcium	42	398.0
Sodium	10.9	49.0
Zinc	3.1	2.3
Iron	8.0	3.3–14.89
Manganese	1.15	17.61–48.43
Copper	1.06	0.47

Source: 15

**Table 3:** Amino acid profiles of different millet grain varieties (pearl and finger millet)

Amino acids (g/100 g)	Pearl millet	Finger millet
Essential amino acid		
Isoleucine	5.1	4.3
Leucine	14.1	10.8
Lysine	0.5	2.2
Methionine	1.0	2.9
Phenylalanine	7.6	6.0
Threonine	3.3	4.3
Valine	4.2	6.3
Histidine	1.7	2.3
Tryptophan	1.2	NA
Nonessential amino acid		
Alanine	8.1	6.1
Arginine	0.9	3.4
Aspartic acid	6.2	5.7
Cystine	0.8	NA
Glutamic acid	22.8	23.2
Glycine	0.7	3.3
Serine	5.4	5.3
Tyrosine	2.7	3.6
Proline	8.2	9.9

Source: 15

Furthermore, [10] reported that the amino acids profile of pearl millet has more lysine, threonine, methionine, and cysteine than sorghum and corn proteins, but is comparable to wheat, barley, and rice [32, 33]. Moreover, pearl millet grain is considered to be similar to maize in terms of its distribution of proteins, particularly true prolamins, which are believed to be soluble in alcohol [34]. Furthermore, [34] reported high level of essential amino acid balance and approves of pearl millet as an important source of energy and proteins for humans. In addition, when comparing the digestibility among the essential amino acids, the following were found to have higher digestibility in pearl millet than corn: arginine, threonine, valine, isoleucine, and leucine. Finger millet is comparatively balanced in its content of essential amino acids because it contains more lysine, threonine, and valine than other millet varieties [13].

### Dietary fibre

Fibre is considered important for gut health as stated by [35] and moderate intake of high fibre in foods could result in an improvement of gut health. Equally, fibre is important in the prevention of heart disease, colon cancer and diabetes [36]. The high dietary fibre content in pearl millet, which is 8% to 9% [32], improves bowl movement. In addition, because of its low digestion features, it increases the transit time which reduces the rate of glucose in the blood; this helps the non-insulin-dependent diabetic patients. [37] reported lesser incidence of diabetes in people who eat millet. Moreover, fibre in millet may help reduce harmful cholesterol, while boosting valuable cholesterol. It also prevents the secretion of bile acids, which causes gallstones in the body [8]. Furthermore, pearl millet, with its high fibre content, helps slow down the movement of food from the stomach to the intestines. This leads to longer food interval durations, which in turn prevents obesity. On the other hand, neutral detergent fibre (NDF) and acid detergent fibre (ADF) in pearl millets are reported by [38] to be about 140 and 62 g/kg, respectively. The levels of ADF and NDF are critical because they impact animal productivity and digestion in livestock production. Thus, the NDF and ADF is higher in finger than pearl millets, validating that pearl millets can be used in poultry feeds since chickens are unable to digest fibrous feedstuffs. Higher ADF and NDF contents in the feed ingredient are simply an indication of low energy, which was the case in finger millets.

### Lipids

The content of fat in pearl millet is estimated to be about 5–7% [39] in comparison to 3.21–7.71% in maize [40]. In addition, [41] reported the percentage of lipid in finger and pearl millet to be 1% and 5%, respectively. Fatty acids such as palmitic, stearic, and linoleic acids are high in pearl millet, whilst oleic acid is found to be a low percentage in comparison to corn [10]. The overall lipid content in pearl millet grain ranges from 1.5 to 6.8%, which is higher than other millet varieties [9]. The free and bound lipid contents of pearl millet range from 5.6 to 6.1 and 0.6 to 0.9%, respectively. With triglycerides, diglycerides and monoglycerides have been identified as free and bound non-polar lipid components in pearl millet [33]. Sridhar and Lakshminarayana [43] observed the total lipid content in finger millet to be 5.2% (2.2% free lipids, 2.4% bound lipids, and 0.6% structural lipids). On the other hand, the major fatty acids recorded in finger millet were observed to be oleic, followed by palmitic and linoleic acids [43]. Saturated fatty acid accounts for 25.6% of finger millet’s total fatty acids profile, while unsaturated fatty acid accounts for 74.4% [43].

### Macronutrients

Millet grain types vary in mineral composition as summarised in Table 2. Martínez-Ballesta [44] states that environmental stressors such as high salt levels, low water accessibility, and excessive temperatures, are found to affect the mineral content of food. Pearl millet consists of total mineral and trace elements, which is determined by the nature of soil. The content of ash in both pearl millet and corn ranges from 1.6 to 3.6% and 0.86 to 1.35%, respectively. With high concentrations of minerals such as calcium, phosphorus, magnesium, manganese, zinc, iron, and copper in pearl millet more than in corn [10]. Pearl millet is also considered a decent resource for fat-soluble vitamin E (2 mg/100 g) owing to its substantial oil content. The grain is also considered a good source of vitamin A [9]. Florence et al. [45], puts the calcium content of pearl millet at 45.6 and 48.6 mg/100 g. It also has a large amount of phosphorus, which is an important mineral in the mineral matrix of bone, adenosine triphosphate or ATP, which is the energy booster in the body. It also helps in bone growth development and repair. However, some studies suggest that pearl millet has high amounts of iron together with many other factors such as phytates, oxalates and polyphones, which may interfere with the bioavailability of iron [46].

Finger millet on the other hand is rich in calcium and ranges from 162 mg/100 g to 487 mg/100 g, depending on the genotypes. [47] In addition, [48] recorded the calcium content of finger millet to be between 189.93 mg per a hundred grammes (mg/100 g) to 1272.36 mg/100 g. Further to this, [49] recorded the calcium in pearl millet to be at the range of 31.77 and 728.71 mg/kg. Furthermore, report by [50] puts the calcium content in finger millet to be 344 mg/100 g. In addition*,* millets contain generous amount of magnesium, which is believed to have the ability to aid the body in fighting diseases such as cancer. Bachar et al. [48] reported a magnesium content of 84.71 mg/100 g to 567.45 mg/100 g in finger millet. Moreover, finger millet is considered a good source of natural calcium, which helps in bone strengthening and reducing the risk of bone fractures.

### Health benefits of finger and pearl millets

Millet is consumed raw with multiple health benefits. It can also be transformed into fermented spinoffs, which are believed to add more benefits to human and animal health. Research shows that diets rich in plant food can protect against different kinds of diseases [51]. It is suggested by different authors that the presence of certain nutrients in millets lead to double benefit of nourishment and cure. Table 4 shows different phenolic compounds present in finger millet, their functions and health benefits.

**Table 4:** Phenolic compounds present in finger millet and their functions

Health compounds	Functions	References
Ferulic acid	Prevents tissue damage and stimulates the wound healing process	[63]
Phytic acid	Plays important role in lowering body cholesterol	[15, 63, 64]
Phenols, phytates, and tannins	Critical in curing aging and metabolic disorders. Inhibits the worsening of human well-being, cancer, and cardiovascular illnesses. Lowering of blood pressure and diabetes. Reduces tumours	[65, 66]
Dietary fibre	Vital for hypoglycemic and hypolipidemic effect as well as cutting of serum cholesterol. Inhibits atherosclerosis and has an antitoxic effect and anti-cancerous effect Energy diluents to formulate low calorie diets	[66, 67] [68]
Nutraceutical foods	Promotes better health by reducing the risk of chronic diseases such as obesity. Lowers blood pressure, cancer, and diabetes	[63]
Magnesium	Reduces the risk of heart attacks	[64]
Phosphorus	Vital for the growth of body tissue and energy metabolism	[64]

Source: 71

### Polyphenols

The main polyphenols such as phenolic acids and tannins are found in abundance in millet and are believed to act as antioxidants and play a vital role in boosting the body’s immune system [52]. In addition, the coat of a finger millet seed, which contains phenols, has an antibacterial effect on Bacillus cereus [53]. Moreover, [37] indicate that millet phenolics can partially inhibit the enzymatic hydrolysis of complex carbohydrates, and consequently, inhibit malt amylase, α-glucosidase, and pancreatic amylase, which reduce postprandial hyperglycaemia. In a similar way, it is believed that ferulic and p-coumaric acids, which are found in whole pearl millet, have the capacity to reduce tumour cells [54]. In addition, [55] reported that the phenols available in millets have numerous health benefits and can act as an antioxidant, anti-inflammatory, and antiviral. Furthermore, celiac disease is a genetically susceptible problem triggered by the consumption of gluten. As the millets are gluten free, they help reduce celiac disease by reducing the irritation caused by common cereal grains that contain gluten. [56].

Millets contain phenolic acids that occur in a bound form (60%) as free molecules. The most common phenolics in millets are hydroxycinnamic acids and are mostly found in the insoluble-bound sections of phenolic acids [57]. The most common type of hydroxycinnamic acid is ferulic acid known as an antioxidant. Antioxidants are known nutrients that help minimise free radical damage to the body, and also have anti-inflammatory activity [58]. In addition, ferulate dimers have been found in millet grains and display high antioxidant activity [59]. Cereal ferulic acids have strong antioxidant activity in bound form, thus do not necessitate microbial activity during digestion to enable their release within the colon [60]. Furthermore, millet grains contain numerous flavonoids, comprising anthocynidins, chalcones, aminophenolics, flavanols, flavones, and flavanones [59]. Furthermore, different millet varieties are also believed to contain proanthocyanidins, also termed condensed tannins as reported by [61]. Significant levels of tannin are mostly observed in coloured millet varieties [17]. This finding was attributed to availability of condensed tannins since they contribute substantially to the grains colour. However, an abundance of condensed tannins may adversely affect the bioavailability of proteins and minerals [62].

### Antioxidant properties of millet

The generous content of phenolic compounds in millet has made it a potent source of antioxidants [61, 72]. Millet grains contained several natural occurring phenolic compounds which include phenolic acids, flavonoids, and tannins, in addition to xylo-oligosaccharides, insoluble fibres and peptides [57]. Table 5 shows compounds and antioxidant properties of pearl and finger millets grains.

**Table 5:** Compounds and antioxidant properties of pearl and finger millets

Millet type	Active compounds	Antioxidant property	References
Finger millet	Phenolic acids	Free radical scavenging, anti-inflammatory activity	[57]
Finger millet	Phenolic compounds	High reducing power (reduction of the ferricyanide to ferrocyanide)	[70]
Pearl millet			
Finger millet	Flavonoids	inhibition of α-glucosidase and α-amylase activities Reduction of postprandial hyperglycemia	[71]
Finger millet	Carotenoid	Quenching of single oxygen and free radicals	[72]
Pearl millet	Phenolic acids	Metal chelating activity	[73]
Pearl millet			

### Anti-nutritional factors present in finger and pearl millets

Anti-nutritional factors are substances that reduce the availability of nutrients when present in animal feed [74]. Their presence in pearl and finger millets is believed to limit protein and starch digestibility, hamper mineral bioavailability, and hinder proteolytic and amylolytic enzymes. Pearl millet is free of gluten and has a low glycaemic index, but despite all the positive characteristics, the antinutrients such as phytic acid, polyphenols, and tannins, can limit its functions as food or feed [75]. Different cultivars of pearl millet contain anti-nutritional factors as phytates [23]. Studies by [76], showed that pearl millet contains 354–796 mg/g^−1^ of phytic acid. Phosphorus in this form is not bioavailable to monogastric because it lacks the digestive enzyme phytase, which is essential when phosphorus is separated from the phytate molecule [77]. In addition, the presence of some goitrogenic polyphenols and C-glycosylflavones, which includes glucosyl vitexin, glucosyl orientin and vitexin, may be responsible for some health problems when pearl millet is consumed [78]. Epidemiologic evidence suggests that millet diets, in rural villages in Africa and Asia, have a role to play in the prevalent development of goitre [75].

On the other hand, finger millet also has its share of the antinutritional factors that include tannins, protease inhibitors, oxalates and phytates, which are believed to inversely affect the digestibility of nutrients [79]. In addition, [50], it has been reported that the presence of anti-nutritional compounds such as phytates, phenols, and tannins in finger millet, have a negative effect on the utilisation of nutrients. The antinutrient tannin is believed to decrease feed intake and feed efficiency [79]. The amount of tannin is believed to be the highest in finger millet in comparison to other millets, which ranges from 0.04 to 3.74% of the catechin equivalents [80]. Furthermore, the antinutrients found in finger millet are believed to vary under different environmental conditions as observed by [81].

Fortunately, the proportion of these antinutritional factors can be reduced by applying different processing methods. Numerous processing techniques such as dehulling, milling, malting, blanching, parboiling, acid and heat treatments, and the fermentation of some forms of pearl millet, seem to reduce the antinutrient factor [82, 86]. Sharma and Kapoor [84], found that germination and debranning coupled with autoclaving, have been proven to be effective in reducing phytic acid and polyphenols. Similarly, [85] found that antinutritional factors can be reduced to a limited amount through the application of various processing techniques such roasting, soaking, boiling, parboiling, fermentation, milling, germination, decortications, and extrusion.

### Millets as food and feed

Millet grains are considered unique crops because they are rich in valuable nutrients such as calcium, dietary fibre, polyphenols, and protein [55]. They are a staple food source for many Asian and African countries. Most of the millet produced is mainly for human consumption, and a lesser percentage is used for livestock and bird feed and beer production [86]. Millet is made into a thin or thick consistency porridge in some parts of Africa, while in other areas it is made into a product called couscous [86]. Research was conducted using the whole grain or crushed grain, which was incorporated in chicken feeds confirming that pearl millet is an effective feed ingredient for poultry production [87]. Pearl millet grain is considered the main purpose for cultivation in Africa and Asia, and the forage is also an important secondary product used for animal feed, fuel, and construction [88]. While season-specific crops such as wheat and rice only provide food security, all-season crop millets ensure food security, fodder, nutrition, health, and sustained livelihood [89]. Pearl millet grains have a high potential as a food source for humans because they are gluten-free and higher in dietary fibre content than rice. They also have the same amount of lipid as found in maize cereal, and they have higher essential amino acids such as leucine, isoleucine and lysine than found in traditional cereals such as wheat and rye [90].

In India, where millet is often used, it is made into dosa, which is a flat bread made of mixture of millet and other grains. It is also made into couscous, cookies, sushi, no yeast pizza and roti [91]. Madua, which is a popular finger millet-based beverage in India, is another product made using millet. In addition, Oshikundu, which is a traditional Namibian alcoholic or non-alcoholic drink, is made from millet [92].

### The use of millet grains in chicken diets

The inclusion of millet grains in animal feed has gained momentum in recent years. A study by [87] has shown that whole pearl millet grain can be included in the broiler diet by up to 50% without it having a negative effect on performance. Another study by [87] indicated that pearl millet varieties have produced comparable results to corn regarding metabolisable energy and digestible amino acids. Similarly, [93] confirmed that the replacement of corn with pearl millet in broilers’ diets has led to significant enhancements of growth and feed efficiency.

Likewise [94] found that the replacement of corn with sorghum and millet by up to 50% of layers’ diets, had similar effects on the egg production rate. Rao et al. [95], found that it can replace corn in broilers’ diets without affecting their body weight gain. In addition, feeding pearl millet to laying hens is believed to have additional benefit because the eggs contain higher omega-3 fatty acids and lower omega-6 [96].

Furthermore, a study by [97] that broilers fed diets containing 25 or 50% millet, had a body weight equal to the fed maize. The males were fed the 25% millet diet and had a higher carcass yield than the feed containing only maize. This clearly indicates that millet can be a substituted for maize. Similarly, in a study by [98], it was suggested that pearl millet could replace 25–50% of maize in a broiler’s diet without affecting its performance. A summary of different inclusion levels of millets and their effect on chicken production is shown in Table 6.

**Table 6:** Responses to the replacement of maize with different inclusion levels of millet regarding feed intake, feed conversion ratio, and body weight of chickens

Study/millet	Millet inclusion level	Feed intake response (g/bird)	Body weight response (g/bird)	Feed conversion ratio
[99]	100%	3949.26	2167.7	2.24
[11]	25%	8216.0	1177.0	6.70
	50%	8461.0	1178.0	7.22
	75%	8547.0	1167.0	5.15
	100%	7864.0	1178.0	5.82
[100]	100%	1177.40	2451.0	2.41
[101]	100%	3030.0	1660.0	2.08
[87]	25%	4368.0	3150.0	1.79
	50%	4452.0	3038.0	1.86
	75%	4494.0	2992.0	1.93
[94]	50%	9540.0	1061.0	Ns
	100%	9450.0	1062.0	Ns
[102]	25%	3634.60	2012.50	1.82
	50%	3628.30	2049.10	1.78
	75%	3609.30	2009.4 0	1.80

*Ns* not specified

### The use of millet grains in the ruminants’ diets

Millet grain and in this case, the finger and pearl millets were evidently used to replace conventional grains, in the feed of small ruminants. An early study conducted by [103] on lactating and growing goats, found that the feed intake and milk production, were not affected upon the replacement of corn with pearl millet. However, they have recorded a depression on the daily growth rate and feed to gain ratio when corn was completely replaced with the pearl millet. They concluded that although pearl millet could be a useful source of alternative energy feed for mature goats, it might not be useful for the growing goats. It is worth noting that the bulk of the studies on millet as a replacement for maize, were conducted on poultry in comparison to the ruminants. Pearl millet was the most used in these studies, probably for the reasons that it is the most popular among the millets and also its superior nutritional values. As stated by [97], pearl millet has proven itself to be equivalent to corn or even superior.

Millet grain is also considered useful in replacement of maize in the big animals. Study by [104] observed that pearl millet grain could fully replace maize in high supplement diets for confined cattle. Millet is found to be beneficial to the animals, fed whole grain or ground. Several research were conducted to establish the most effective way of feeding of millet. A study by [104] found that processing of millet grain increases the digestibility of dry matter and dietary nutrients of grazing beef cattle during dry season. Table 7 summarises different studies on replacement of maize with millet in ruminant animals.

**Table 7:** Pearl and finger millet inclusion and their effect on ruminant’s performance

Millet type	Inclusion level	Effect on performance	References
Pearl millet		Improved digestibility	[105]
Pearl millet	30%	no adverse effects on milk yield or milk composition	[110]
Pearl millet		Improved average daily gain	[111]
Pearl millet		Similar average daily gain and final body weight as corn	[112]
Pearl millet	50% 100%	Increased digestibility of starch and Ether Extracts Reduced ruminal ammonia concentration	[113]
Pearl millet	79%	Similar performance indicators to those obtained with corn and sorghum	[114]
Pearl millet	25% 50% 75% 100%	No effect on dry matter intake, milk yield and milk fat percent	[115]
Pearl millet	33% 665 100%	Performances were not negatively affected by the substitution of maize with pearl millet	[116]
Finger millet	16.0% 32.5% 48.0% 67.0%	Reduced digestibility of the dry matter No effect on nutrients intake	[117]
Pearl millet	40%	Digestion coefficients for DM, GE, CP, and NDF were reduced by over 10 percentage units with partial or complete replacement of corn by pearl millet	[103]

### Food applications

Millet originate din the African continent, and hence our African ancestors have different by-products that have been produced using millets. The by-products have been passed down to multiple generations. Alcoholic and non-alcoholic have been made using millet. Unfortunately, their composition and the potential benefits for the human body are yet to be explored. Traditional African beverage-making involves several processes, such as soaking, drying, and fermenting. Haggblade and Holzapfel [103] clarify that African beer differs from Western beer because it is sour, less carbonated, and often unrefined. Several African beverages exist; one such beer beverage is togwa, which is a lactic acid fermented beverage [104]. This beverage is mainly found in Tanzania. Bushera*,* which is a lactic acid fermented beverage found in Uganda, can be consumed by adults and children when it is fresh. Masvusvu is a sweet beverage traditionally made from malted finger millet, and is mainly found in Zimbabwe also produces mangisi, which is a sweet–sour product that is produced from the natural fermentation of sieved masvusvu [105]. Marrisa is a local Sudanese alcoholic drink that is made from sorghum or millet using the fermentation method [106]. In Tanzania, cipumu is produced from finger millet and plays an important role in rituals and is a source of for the locals.

In Tanzania, finger millet is utilised as an ingredient to make four types of food, namely, ugali, uji, vtogwa and pombe. In some parts of Tanzania, finger millet is considered the main ingredient in an alcoholic beverage called pombe. Three kinds of pombe is produced using finger millets, namely, kimpumu, komoni and kiambule [107]. The villagers believe that germinated finger millet, gives the pombe a strong taste [107].

In Nigeria, pearl millet is used to make a fried cake called masa*.* The flour is used to make tuwo, which is a thick binding paste [108]. The green fodder is usually fed to the animals. In Zimbabwe, [109] noted that there is an unwillingness from customers when it comes to the use of millet as a basic food source. They attributed this to colour, taste, and flavour of the millet, in addition to general practices and lifestyles of some families. This has led to the farmers not being keen to produce more millet products, and hence, the crop is mainly produced and used for preparing traditional beer brands such as Chibuku [109].

### Challenges of food security in the developing countries

Food security is defined as a situation whereby, people have physical and economic access to safe and nutritious food to meet their dietary requirements [118]. Food security challenges are often narrowed to supply of agricultural produce such as livestock, as pointed out by [119]. However, the challenges are believed to be more complex than just increasing the supplies. Many factors such as urbanisation and accessibility are among the constraints described by [119]. Furthermore, institutional failures as well as structure and processes which are governing economies and societies are also listed as some of the factors which cause food insecurity [120]. In a study by Abegaz, [121], on food security status in Ethiopia, the first rain shock which is a product of the climate change, is considered one of the main impactors of food security. Fraval et al. [122], noted that the casual intervention to prevent food insecurity, is not necessarily straightforward, therefore proxies of interventions and greater understanding of different proposed pathways are important in successful interventions. These complex hindrances require multisectoral approaches and planning to resolve them. According to Pangaribowo, et al. [120], the ideal way to deal with the issues of food and nutrition security is to couple the indicators of food insecurity together with available socioeconomic and environmental indicators of a particular entity. Regardless of the challenges which face the food security in the developing countries, the contribution of underutilised and locally produced grains such as millet cannot be overlooked. The conventional grain crops are not enough to overcome some of these challenges [123]. Tying in situations such as the current unprecedented COVID-19 pandemic, development of minor grains such as millet could elevate poverty among poor population [123]. From the climatic change context, millets are the crops with the potential to survive harsh conditions and contribute to the stability of food security. Padulosi et al. [124], reported that minor millets such as finger, kodo, foxtail, little, proso and barnyard, have the ability to grow successfully in diverse soils, varying rainfall regimes, diverse photoperiods and in marginal, due to their genetic adaptation. These characteristics qualify the millets to replace commodities like wheat and rice in harsh climatic zones, eventually leading to food security in these areas. However, millet is considered a neglected agro-biodiversity, though it has the potential to agricultural system and food security among the poor population in Sub- Saharan Africa [125].

### The cost benefit of using millet

Millet is a gluten-free and low-cost cereal with an estimated cost of 40% lower than corn [126]. Silva et al. [127] has put the trade value of pearl millet to be less than or equal to 77.78% of the cost of the corn grain. The protein content of pearl millet grain is higher than in maize, which may allow formulation of diets without supplementation of protein, consequently reducing the cost of food and feed.

In addition, the cost of producing millet is less than producing other grains such as maize and sorghum. For example, pearl millet water-use is more efficient than sorghum and maize grown in semi-arid regions of Brazil (56 ± 2.8 kg DM/ha/mm (kilogrammes of dry matter per hectare per millimetre) water for the Brazilian pearl millet cultivars v. 45 ± 1.9 kg DM/ha/mm water for sorghum; [128]; and 21 ± 2.4 kg DM/ha/mm water for the Brazilian maize cultivars; [129]. In a study by [126], the total replacement of maize with pearl millet, was found to be the most economical in the diet of feedlot cattle. The items which influenced the financial indicator were reported to be the price of lean and fat cattle, initial weight, final weight, cost of concentrate, cost of roughage, consumption of concentrate and consumption of roughage [130]. It is also logical to assume that positioning of millet as competitive grain to maize, will tilt the weight of the supply, which will consequently relieve the pressure on maize consumption, resulting in price reduction. In another study by [131] reported that the cost of feed required to produce one kg of live weight gain in maize fed group of chickens was higher than in pearl millet, finger millet and sorghum fed groups. Medugu et al. [99], confirmed that it is more economical and cost effective to produce broiler chicken, as the cost per kg feed and cost of feed per unit weight gain are lowest in millet grains feed. Wilson et al. [132], estimated that total net profit from the use of pearl millet as the sole feedstock was $25,175,000 per year compared to $23,758,000 for maize feedstocks, about $1.4 million advantage.

## Conclusions

It has been demonstrated from the studied literature that pearl and finger millets have the potential to be used as an alternative source of energy in poultry diets. It has competitive nutrients equal to, or in some instances more than, conventional cereals such as maize, wheat and rice. In addition, the presence of nutraceuticals in the millets give them extra importance in terms of health benefits, especially for humans. The inclusion of up to 100% of millets can be added to broiler diets without having negative effects on the performance of chickens. Inclusion of millet in ruminants’ animals’ diets also had noticeable improvements on the performance parameters. This inclusion could eventually reduce the cost of feed for livestock production and consequently reduce the cost of livestock products for people who rely on it as source of protein. Because the grain is gluten free, it is considered one of the most suitable grain for the people with celiac diseases. Further to this, millet grains contain antinutrients that can have an adverse impact on nutrient bioavailability. However, different processing methods have been proven to reduce the adverse effects of the antinutrients. Further study is necessary to establish the optimum inclusion level of millets in animal diets. In addition to that, creation of awareness to stress on the importance of these millets for human health is highly encouraged.

## Data Availability

Not applicable.

## Abbreviations

NDF: Neutral detergent fibre
ADF: Acid detergent fibre
mg/100 g): Milligramme per a hundred grammes
kg DM/ha/mm: Kilogrammes of dry matter per hectare per millimetre
FAO: Food and Agriculture Organization

## References

[1] Gari J A. Review of the African millet diversity Paper for the International workshop on fonio, food security and livelihood among the rural poor in West Africa; 2002. http://www.ipgri.org. Accessed 24 Mar 2020.

[2] FAO (food and agriculture organization). World food situation; 2017. http://www.fao.org/worldfoodsituation/csdb/en/. Accessed 25 Feb 2020.

[3] Dube T, Mlilo C, Moyo P, Ncube C, Phiri K (2018). Will adaptation carry the future? Questioning the long-term capacity of smallholder farmers’ adaptation strategies against climate change in Gwanda District. Zimbabwe J Hum Ecol.

[4] Sharma KK, Ortiz R (2000). Program for the application of genetic transformation for crop improvement in the semi-arid tropics. In Vitro Cell Dev Biol Plant.

[5] FAOSTAT. 2014. FAO statistical yearbook. FAO, Rome. http://www.fao.org/3/a-i3590e.pdf. Accessed 12 Feb 2020.

[6] Patel K, Gartaula H, Johnson D (2015). The interplay between household food security and wellbeing among small-scale farmers in the context of rapid agrarian change in India. Agric Food Secur..

[7] Anitha S, Govindaraj M, Kane-Potaka J (2019). Balanced amino acid and higher micronutrients in millets complements legumes for improved human dietary nutrition. Cereal Chem.

[8] Shweta M (2015). Pearl millet nutritional value and medicinal uses. IJARIIE-ISSN (O).

[9] Taylor JRN, Wrigley C, Corke H, Walker CE (2004). Millet: in encyclopaedia in grain science.

[10] Adeola O, Orban JI (1995). Chemical composition and nutrient digestibility of pearl millet (*Pennisetum glaucum*) fed to growing pigs. J Cereal Sci.

[11] Tadele Z. Drought Adaptation in Millets. 2016. http://dx.doi.org/10.5772/61929. Accessed 12 Feb 2020.

[12] Lost Crops of Africa: Volume I: Grains. Washington, DC: The National Academies Press; 1996. 10.17226/2305.

[13] Ravindran G (1991). Studies on millets: proximate composition, mineral composition, and phytate and oxalate contents. Food Chem.

[14] Sobana S, Sreerama YN, Malleshi NG (2009). Composition and enzyme inhibitory properties of finger millet (*Eleusine coracana* L.) seed coat phenolics: mode of inhibition of α-glucosidase and pancreatic amylase. Food Chem.

[15] Amadou I, Gounga ME, Guo-Wei L (2013). Millets: Nutritional composition, some health benefits and processing—a Review. Emir J Food Agric.

[16] Cisse RS, Hamburg JD, Freeman ME, Davis AJ (1996). Board on science and technology for interval development. Lost crops of Southern Africa: Grains.

[17] McDonough CM, Rooney LW, Serna-Saldivar SO, Kurl K, Ponte JG (2000). The millets in handbook of cereal science and technology, second edition, revised and expanded, chap 4.

[18] Abdelrahman A, Hoseney RC, Varriano-Marston E (1984). The proportions and chemical compositions of hand-dissected anatomical parts of pearl millet. J Cereal Sci.

[19] FAO. Annual Publication. Rome, Italy: Food and Agricultural Organization 2007. http://www.fao.org/3/a-a1200e.pdf. Accessed 12 Dec 2019.

[20] McDonough CM, Rooney LW (1989). Structural characteristics of *Pemtisetum americanum* using scanning electron and fluorescence microscopies. Food Microstructure.

[21] Shobana S. Investigations on the carbohydrate digestibility of finger millet (Eleusine coracana) with special reference to the influence of its seed coat constituents PhD Theses, 2009. University of Mysore, Mysore

[22] Rooney LW, Kirleis AW, Murty DS, Pomeranz Y (1986). Traditional foods from sorghum: their production, evaluation and nutritional value. Advances in cereal science and technology.

[23] Ouattara-Cheik AT, Aly S, Yaya B, Alfred TS (2006). A comparative study on nutritional and technological quality of fourteen cultivars of pearl millets Pennisetum glaucum (L) Leek in Burkina Faso. Pakistan J Nutr.

[24] Suma F, Urooj A (2015). Isolation and Characterization of Starch from Pearl Millet (*Pennisetum typhoidium*) Flours. Int J Food Prop.

[25] Hadimani NA, Muralikrishna G, Tharanathan RN, Malleshi NG (2001). Nature of carbohydrates and proteins in three pearl millet cultivars varying in processing characteristics and kernel texture. J Cereal Sci.

[26] Bhatt A, Singh V, Shrotria PK, Baskheti DC (2003). Coarse grains of Uttaranchal: ensuring sustainable food and nutritional security. Indian Farmer’s Digest.

[27] Wankhede DB, Shehnaj A, Rao MR (1979). Carbohydrate composition of finger millet (Eleusine coracana) and foxtail millet (Setaria italica). Plant Foods Hum Nutr.

[28] Jha A, Tripathi AD, Alam T, Yadav R (2013). Process optimization for manufacture of pearl millet-based dairy dessert by using response surface methodology (RSM). J Food Sci Technol.

[29] Burton GW, Wallance AT, Radice KO (1972). Chemical composition during maturation and nutritive value of pearl millet. Crop Sci.

[30] Chethan S, Malleshi NG (2007). Finger millet polyphenols: optimization of extraction and the effect of pH on their stability. Food Chem.

[31] Wafula WN, Ojulong HF, Siamb MI, Gweyi-Onyango JP (2018). Protein, Calcium, Zinc, and iron contents of finger millet grain response to varietal differences and phosphorus application in Kenya. Agron.

[32] Rooney LW, Miller FR. Variation in the structure and kernel characteristics of sorghum. In: proceeding of the international symposium on sorghum grain quality. iCRISAT. 28–31. Patancheru, India. 1982; 143–162.

[33] Hoseney RC (1994). Principles of cereal science and technology.

[34] Ejeta GM, Hassen MM, Mertz ET (1987). In vitro digestibility and amino acid composition of pearl millet (Pennisetum typhoides) and other cereals (pepsin digestibility/protein fractionation/protein quality). Proc Natl Acad Sci USA.

[35] McIntosh GM, Noakes M, Royle PJ, Foster PR (2003). Whole-grain rye and wheat foods and markers of bowel health in overweight middle-aged men. Am J Clin Nutr.

[36] Eshak ES, Iso H, Date C, Kikuchi S, Watanabe Y, Wada Y, Wakai K, Tamakoshi A (2010). Dietary fiber intake is associated with reduced risk of mortality from cardiovascular disease among Japanese men and women. J Nutr.

[37] Shobana S, Sreerama YN, Malleshi NG (2009). Composition and enzyme inhibitory properties of finger millet (*Eleusine coracana* L.) seed coat phenolics: mode of inhibition of α-glucosidase and pancreatic amylase. Food Chem.

[38] Mustafa A, Seguin P, Bélair G, Kumar A (2008). Chemical composition and ruminal degradability of grain pearl millet grown in southwestern Quebec. Can J Anim Sci.

[39] Gopalan C, Rama Sastri BV, Balasubramanian SC (2003). Nutritive value of Indian foods.

[40] Ullah I, Ali M, Farooqi A (2010). Chemical and Nutritional Properties of Some Maize (Zea mays L) Varieties Grown in NWFP. Pakistan. PJN..

[41] Himanshu K, Chauhan M, Sonawane SK, Arya SS (2018). Nutritional and nutraceutical properties of millets: a review. Clin J Nutr Diet..

[42] Sridhar R, Lakshminarayana G (1994). Contents of total lipids and lipid classes and composition of fatty acids in small millets: foxtail (Setaria italica), proso (Panicum miliaceum), and finger (Eleusine coracana). Cereal Chem.

[43] Kunyanga CN, Imungi JK, Velingiri V (2013). Nutritional evaluation of indigenous foods with potential food-based solution to alleviate hunger and malnutrition in Kenya. J Appl Biosci.

[44] Martínez-Ballesta MC, Dominguez-Perles R, Moreno DA, Muries B, Alcaraz-López C et al. Minerals in plant food: effect of agricultural practices and role in human health. A review. Agronomy for Sustainable Development, Springer Verlag/EDP Sciences/INRA, 2010. Doi: 10.1051/agro/2009022.

[45] Florence SP, Asna U, Asha MR, Jyotsna R (2014). Sensory, physical, and nutritional qualities of cookies prepared from pearl millet (Pennisetum typhoideum). J Food Processing Techno.

[46] Nambiar VS, Dhaduk JJ, Sareen N, Shahu T, Desai R (2011). Potential functional implications of pearl millet (*Pennisetum glaucum*) in health and disease. J Appl Pharm Sci.

[47] Vadivoo AS, Joseph R, Ganesan NM (1998). Genetic variability and calcium contents in finger millet (Eleusine coracana L. Gaertn) in relation to grain colour. Plant Foods Hum Nutr.

[48] Bachar K, Mansour E, Ben Khaled A, Abid M, Haddad M, Ben Yahya L, Jarray EL, N, Ferchichi A.  (2013). Fiber content and mineral composition of the finger millet of the oasis of Gabes Tunisia. J Agric Sci..

[49] Adéoti K, Kouhoundé SHS, Noumavo PA, Baba-Moussa F, Toukourou F (2017). Nutrional vale and physiological composition of pearl millet (*PENNISETUM GLAUCUM*) produced in Benin. J Microbiol Biotechnol Food Sci.

[50] Singh P, Raghuvanshi RS (2012). Finger millet for food and nutritional security. African J Food Sci.

[51] Chandrasekara A, Shahidi F (2012). Bioaccessibility and antioxidant potential of millet grain phenolics as affected by simulated in vitro digestion and microbial fermentation. J Funct Foods.

[52] Chandrasekara A, Shahidi F (2010). Content of insoluble bound phenolics in millets and their contribution to antioxidant capacity. J Agric Food Chem.

[53] Viswanath VA, Urooj A, Malleshi NG (2009). Evaluation of antioxidant and antimicrobial properties of finger millet polyphenols (*Eleusine coracana*). Food Chem.

[54] Chandrasekara A, Shahidi F (2011). Determination of antioxidant activity in free and hydrolyzed fractions of millet grains and characterization of their phenolic profiles by HPLC-DAD-ESI-MSn. J Funct Foods.

[55] Devi PR, Vijayabharathi R, Sathyabama S, Malleshi NG, Priyadarisini VB (2014). Health benefits of finger millet (Eleusine coracana L.) polyphenols and dietary fibre: a review. J Food Sci Technol.

[56] Saleh ASM, Zhang Q, Chen J, Shen Q (2013). Millet grains: nutritional quality, processing, and potential health benefits. Compr Rev Food Sci Food Saf.

[57] Liang S, Liang K (2019). Millet grain as a candidate antioxidant food resource: a review. Int J Food Prop.

[58] Lobo V, Patil A, Phatak A, Chandra N (2010). Free radicals, antioxidants and functional foods: Impact on human health. Pharmacogn Rev.

[59] Emiola LO, De la Rosa LC (1981). Characterization of Pearl Millet Nonstarchy Polysaccharides. J Food Sci.

[60] Călinoiu LF, Vodnar DC (2018). Whole grains and phenolic acids: a review on bioactivity, functionality, health benefits and bioavailability. Nutrients.

[61] Dykes L, Rooney LW (2006). Sorghum and millet phenols and antioxidants. J. Cereal Sci.

[62] Chavan UD, Shahidi F, Naczk M (2001). Extraction of condensed tannins from beach pea (Lathyrus maritmus L.) as affected by different solvents. Food Chem.

[63] Sarita ES, Singh E. Potential of millets: nutrients composition and health benefits. j. sci. innov. res. 2016; 5(2):46–50. Retrieved from https://www.jsir.journal.com. Accessed 13 April.

[64] Chandra A, Singh AK, Mahto B. Processing and value addition of finger millet to achieve nutritional and financial security—case study. IJCMAS. 2018; 7, 2901–2910. Retrieved from https://www.ijcmas. Com. Accessed 15 Jan 2020.

[65] Siwela M, Taylor JRN, de Milliano WAJ, Doudu KG (2007). Occurrence and location of tannins in finger millet grain and antioxidant activity of different grain type. Cereal Chem.

[66] Thilagavathi T, Banumathi P, Kanchana S, Ilamaran M (2015). Effect of heat moisture treatment on functional and phytochemical properties of native and modified millet flours. Plant Arch.

[67] Udeh HO, Doudu KG, Jideani AIO (2017). Finger millet bioactive compounds, bioaccessibility, and potential health effects a review. Czech J Food Sci.

[68] Rao D B, Bhaskarachary K, Christina A GD, Devi S G, Vilas A. Tonapi VA. 2017. Nutritional and Health benefits of Millets. ICAR_Indian Institute of Millets Research (IIMR) Rajendranagar, Hyderabad, PP 112.

[69] Shahidi F, Chandrasekara A (2013). Millet grain phenolics and their role in disease risk reduction and health promotion: a review. J Funct Foods.

[70] Kumari D, Madhujith T, Chandrasekara A (2017). Comparison of phenolic content and antioxidant activities of millet varieties grown in different locations in Sri Lanka. Food Sci Nutr.

[71] Ofosu KF, Elahi FY, Daliri EB, Chelliah R, Ham HJ, Kim J, Han JS, Oh D (2020). Phenolic profile, antioxidant, and antidiabetic potential exerted by millet grain varieties. Antioxidants.

[72] Viswanath V, Urooj A, Malleshi NG (2009). Evaluation of antioxidant and antimicrobial properties of finger millet polyphenols (Eleusine coracana). Food Chem.

[73] Jayalaxmi R, Divya K, Ankita S, Koteswaraiah P (2018). Antioxidant activities of Pearl millet (*Pennisetum glaucum*) and Little millet (*Panicum sumatrense*) in different *in vitro*models. J Bioassays.

[74] Yacout MHM (2016). Anti-nutritional factors & its roles in animal nutrition. J Dairy Vet Anim Res.

[75] Boncompagni E, Orozco-Arroyo G, Cominelli E, Gangashetty PI, Grando S, Kwaku Zu TT, Daminati MG, Erik Nielsen E, Sparvoli F (2018). Antinutritional factors in pearl millet grains: Phytate and goitrogens content variability and molecular characterization of genes involved in their pathways. PLoS One.

[76] Abdalla AA, Elinay AH, Mohamed BE, Abdallah AH (1998). Proximate composition, starch, phytate and mineral contents of ten pearl millet genotypes. Food Chem.

[77] Pelig-Ba KB (2009). Assessment of phytic acid levels in some local cereal grains in two districts in the upper east region of Ghana. Pak J Nutr.

[78] Gaitan E, Lindsay RH, Reichert RD, Ingbar SH, Cooksey RC, Legan J (1989). Antithyroid and goitrogenic effects of millet: role of C-glycosylflavones. J Clin Endocrinol Metab.

[79] Kumar SI, Babu CG, Reddy VC, Swathi B (2016). Anti-nutritional factors in finger millet. J Nutr Food Sci.

[80] Ramachandra G, Virupaksha TK, Shadaksharaswamy M (1977). Relationship between tannin levels and *in vitro* protein digestibility in finger millet (*Eleusine coracana* Gaertn). J Agric Food Chem..

[81] Shibairo SI, Nyongesa O, Onwonga R, Ambuko J (2014). Variation of nutritional and anti-nutritional contents in finger millet (Eleusine coracana (L.) Gaertn) genotypes. SJAVS..

[82] Eltayeb MM, Hassn AB, Sulieman MA, Babiker EE (2007). Effect of processing followed by fermentation on antinutritional factors content of pearl millet (Pennisetum glaucum L.) cultivars. Pak J Nutr.

[83] Zhang L, Li J, Han F, Ding Z, Fan L (2017). Effects of different processing methods on the antioxidant activity of 6 cultivars of foxtail millet. J Food Qual.

[84] Sharma A, Kapoor AC (1996). Levels of antinutritional factors in pearl millet as affected by processing treatments and various types of fermentation. Plant Food Hum Nutr.

[85] Rathore T, Singh R, Kamble DB, Upadhyay A, Thangalakshmi S (2019). Review on finger millet: processing and value addition. J Pharm Innov..

[86] Obiana AB. Overview: importance of millets in Africa 2003. Published online at http://www.afripro.org.uk/papers/Paper02Obil. Accessed 7 Feb 2020.

[87] Cisse RS, Hamburg JD, Freeman ME, Davis AJ (2016). Using locally produced millet as a feed ingredient for poultry production in Sub-Saharan Africa. J Appl Poultry Res.

[88] Andrews DJ, Kumar KA (1992). Pearl millet for food, feed, and forage. Adv Agron.

[89] Shivananjappa M (2018). Meats are malady and millets are magical. J Nutr Food Technol.

[90] ICRISAT. Smart food millet recipes 2016. http://www.icrisat.org/PDF/Food-Booklet-Millet.pdf. Accessed 16 May 2020.

[91] Dias-Martins AM, Kênia Letícia P, Sidney P, José Avelino SR, Carlos WPC (2018). Potential use of pearl millet (Pennisetum glaucum (L.) R. Br.) in Brazil: food security, processing, health benefits and nutritional products. Food Res Int.

[92] Kumar A, Tomer V, Kaur A, Kumar V, Gupta K (2018). Millets: a solution to agrarian and nutritional challenges. Agric Food Secur.

[93] Baurhoo NB, Baurhoo AF, Mustafa ZX (2011). Comparison of corn-based and Canadian pearl millet-based diets on performance, digestibility, villus morphology, and digestive microbial populations in broiler chickens. Poult Sci.

[94] Issa S, Jarial S, Brah N, Harouna L (2016). Are millet and sorghum good alternatives to maize in layer’s feeds in NIGER, West Africa. Indian J Anim Sci.

[95] Rao SVR, Raju MVLN, Reddy MR, Panda AK (2004). Replacement of yellow maize with pearl millet (*Pennisetum typhoides*), foxtail millet (*Setaria italica*) or finger millet (*Eleusine coracana*) in broiler chicken diets containing supplemental enzymes. Asian-Aust J Anim Sci.

[96] Jacob J. Feeding pearl millet to poultry 2015. https://articles.extension.org/pages/68861/feeding-pearl-millet-to-poultry.

[97] Davis AJ, Dale NM, Ferreira, FJ. Pearl millet as an alternative feed ingredient in broiler diets. Poult Scie 2003. Association, Inc.

[98] Tornekar AP, Munde VK, Kokane SS (2009). Effect of replacing maize with bajra (pearl millet) on the performance of broilers. Vet World.

[99] Medugu CI, Kwari ID, Igwebuike J, Nkama I, Mohammed ID, Hamaker B (2010). Performance and economics of production of broiler chickens fed sorghum or millet as replacement for maize in the semi-arid zone of Nigeria. Agric Biol J North Am.

[100] Bulus E, Ibe E, Dodo S, Makinde ISO (2014). Performance of broiler chickens fed two varieties of guinea corn and millets as replacement for maize. Iran J Appl Anim Sci.

[101] AL-Shwilly HAJ, Jiheel MJ, Seger DK (2019). Impact of complete replacement of corn by millet with enzymes in broilers diet on some physiological parameters and performance. Plant Arch.

[102] Bala S, Sharma RK, Khanl N, Rastogi A, Haq Z (2017). Performance of broiler chicken as affected by replacement of maize with pearl millet and broken rice mixture in the diet. Indian J Anim Nutr.

[103] Haggblade S, Holzapfel H, Streinrous KH (2004). Industrialization of Africa’s indigenous beer brewing. Industrialization of indigenous fermented foods.

[104] Mugula JK, Nnko SAM, Narvhus JA, Sorhaug T (2003). Microbiological and fermentation characteristics of togwa, a Tanzanian fermented food. Int J Food Microbiol.

[105] Zvauya R, Mygochi T, Parawira W (1997). Microbial and biochemical changes occurring during production of masvusvu and mangisi, traditional Zimbabwean beverages. Plant Foods Hum Nutr.

[106] Dirar HA (1994). Commentary: the fermented foods of the Sudan. Ecol Food Nutr.

[107] Kubo R (2016). The reason for the preferential use of finger millet (Eleusine coracana) in eastern African brewing. J Inst Brew.

[108] Izge AU, Song IM. Pearl millet breeding and production in Nigeria: problems and prospects. journal of environmental issues and agriculture in developing countries 2013; 5(2).

[109] Phiri K, Dube T, Moyo P, Ncube C, Ndlovu S, Buchenrieder G (2019). (Reviewing editor) (2019) Small grains “resistance”? Making sense of Zimbabwean smallholder farmers’ cropping choices and patterns within a climate change context. Cogent Soc Sci.

[110] Mustafa AF (2009). Short communication: performance of lactating dairy cows fed pearl millet grain. J Dairy Sci.

[111] Hill GM, Hanna WW (1990). Nutritive characteristics of pearl millet grain in beef cattle diets. J Anim Sci.

[112] Alencar WM, Restle J, Missio RL, Neiva JNM, Miotto FR, Freitas IB (2015). Feeding behavior and productive performance of steers fed pearl millet grain-based diets containing proportions of babassu mesocarp bran. R Bras Zootec.

[113] Goncalves JR, Alexandre VP, Ivanete S, laisse GL, Clayton QM, Evndro MF (2010). Replacement of corn grain by pearl millet grain in diets containing corn or elephant grass silage fed beef cattle. R Bras Zootec.

[114] Hill GM, Newton GL, Streeter MN, Hanna WW, Utley PR, Mathis MJ (1996). Digestibility and utilization of pearl millet diets fed to finishing beef cattle. J Anim Sci.

[115] Ribeiro CD, Pires AV, de Simas JM, Santos FA, Susin I, Junior RC (2004). Substituição do grão de milho pelo milheto (Pennisetum americanum) na dieta de vacas holandesas em lactação. R Bras Zootec.

[116] Alonso MP, de Moraes EHB, Pereira DH, dos Santos PD, Mombach MA, Hoffmann A, De Moura GB, Sanson RM (2017). Pearl millet grain for beef cattle in crop-livestock integration system: intake and digestibility. Semina Ciências Agrárias Londrina.

[117] Dos Santos JW, Cabral LS, Zervoudakis JT, de Souza AL, de Abreu JG, Reverditto R, Pereira GA (2008). Finger millet grain levels in sheep diets: intake and digestibility. R Bras Zootec.

[118] World Food Summit. The world free from hunger. Rome; 1996

[119] Hatab AA, Cavinato MER, Lagerkvist CJ (2019). Urbanization, livestock systems and food security in developing countries: a systematic review of the literature. Food Secur.

[120] Pangaribowo, EH, Gerber, N, & Torero, M. Food and nutrition security indicators: a review. *FOODSECURE working paper 04.* 2013; https://www.wecr.wur.nl/WECRGeneral/FoodSecurePublications/05_Pangaribowo%20Gerber%20Torero_FNS%20Indicators.pdf. Accessed on 23^rd^ of November 2020.

[121] Abegaz KH (2017). Determinants of food security: evidence from Ethiopian Rural Household Survey (ERHS) using pooled cross-sectional study. Agric & Food Secur.

[122] Fraval S, Yameogo V, Ayantunde A (2020). Food security in rural Burkina Faso: the importance of consumption of own-farm sourced food versus purchased food. Agric & Food Secur.

[123] Muthamilarasan M, Prasad M. small millets for enduring food security amidst pandemics. https://doi.org/10.1016/j.tplants.2020.08.008. Accessed 23 Nov 2020 10.1016/j.tplants.2020.08.008 PMC7474701 32900620

[124] Padulosi S, Mal B, Ravi SB, Gowda J, Gowda KTK, Shanthakumar G, Yenagi N, Dutta M (2009). Food security and climate change: role of plant genetic resources of minor millets. Indian J Plant Genet Resour.

[125] Garí, JA. Review of the African millet diversity http://www.fao.org/fileadmin/templates/esw/esw_new/documents/Links/publications_other/6_millets.pdf. Accessed 23 Nov 2020.

[126] Gomes PC, Rodrigues MP, Albino LFT, Rostagno HS, Gomes MFM, De Mello HH, Brumano G (2008). Determination of chemical composition and energy value of millet and their use in rations of broilers from 1 to 21 days of age. Rev Bras Zootec.

[127] Silva GAH, Restle J, Missio RL, Bilego UO, Fernandes JJR, Rezende PLP, Medeiros Da Silva R, Pereira MLR, Lino FA (2014). Milheto em substituição ao milho na dieta de novilhos confinados. Semina Ciências Agrárias.

[128] Silva DA, Santos TC, Azevedo EM, Edvan JAG, Perazzo RL, Pinho AF, Rodrigues RMA, DA SILVA, D. S.  (2011). Agronomic divergence of sorghum hybrids for silage yield in the semiarid region of Paraiba. R Bras Zootec.

[129] Dos Santos RD, Pereira LGR, Neves ALA, Azevedo JAG, Demoraes SA, Costa CTF (2010). Agronomic characteristics of maize varieties for silage production in the submédio São Francisco river valley. Acta Sci Anim Sci.

[130] Silva RM, João Restle RZ, Fabricio EA, Camera A, Maysonnave GS, Ubirajara O, Bilego UO, Pacheco PS, Vaz FN (2020). Economic analysis of the risk of replacing corn grains (*Zea mays*) with pearl millet grains (*Pennisetum glaucum*) in the diet of feedlot. Cienc Rural.

[131] Rama Rao SVG, Shyam Sundar AK, Panda MR, Reddy MV, Raju LN, Praharaj NK (2002). Utilization of different millets replacing maize in coloured broiler chicken diet. Indian J Anim Nutr.

[132] Wilson JP, McAloon AJ, YeeW, McKinneyJ, WangD, BeanSR. Biological and economic feasibility of pearl millet as a feedstock for ethanol production. 2007; http://citeseerx.ist.psu.edu/viewdoc/download?doi=10.1.1.485.9513&rep=rep1&type=pdf

